# Perfluoro Allyl Fluorosulfate (FAFS): A Versatile Building Block for New Fluoroallylic Compounds

**DOI:** 10.3390/molecules16086512

**Published:** 2011-08-04

**Authors:** Ivan Wlassics, Vito Tortelli, Serena Carella, Cristiano Monzani, Giuseppe Marchionni

**Affiliations:** Solvay Solexis, Viale Lombardia, 20-Bollate, Milan 20021, Italy; Email: vito.tortelli@solvay.com (V.T.); serena.carella@solvay.com (S.C.); cristiano.monzani@solvay.com (C.M.); giuseppe.marchionni@solvay.com (G.M.)

**Keywords:** perfluoroallyl fluorosulfate, addition/elimination reactions, fluorinated allyl ethers, perfluoro-diallyl peroxide, pK_a_

## Abstract

In this study we will present and discuss both the synthesis of CF_2_=CFCF_2_OSO_2_F (perfluoroallyl fluorosulfate, FAFS), focusing in particular on the important role of C_3_F_6_/SO_3_ ratio, reaction temperature and boron catalyst/SO_3_ ratio on FAFS’ yield and selectivity, as well as a wide variety of ionic and radical reactions possible with FAFS. We focused our attention on reactions of FAFS with aliphatic and aromatic alcohols, acyl halides, halides, H_2_O_2_, ketones and radicals whose synthesis and reaction mechanisms will be presented and discussed. Particular attention will be devoted to the novel diallyl-fluoroalkyl peroxide obtained. Factors such as pK_a_ and Lowry and Pearson’s Hard/Soft Acid-Base Theory which determine the selectivity between Addition/Elimination *vs.* Nucleophilic Substitution reaction mechanisms on FAFS will also be presented and discussed.

## 1. Introduction

**Scheme 1:**
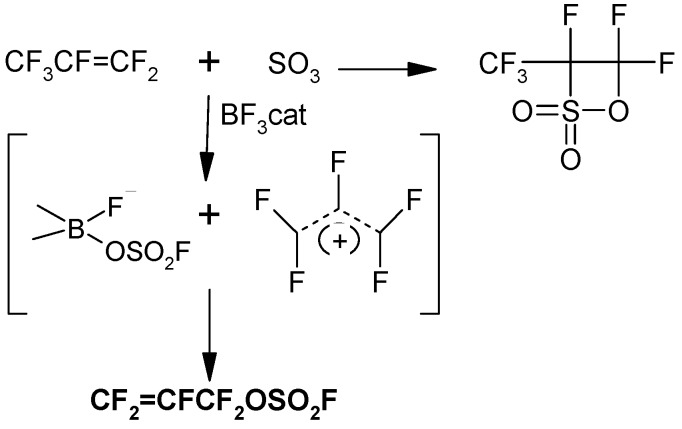
BF_3_ mediated synthesis of FAFS *vs.* synthetic route in the absence of BF_3_.

Early literature studies of fluoro olefin reactions with sulfur trioxide (SO_3_) have shown that the principal reaction of terminal fluoro olefins is a [2+2] cycloaddition to form sultones [1,2]. If the SO_3_ employed in the reaction with hexafluoropropene contains as low as 0.5 wt % of a boron-based catalyst (sometimes used to stabilize commercial SO_3_: Sulfan^®^): BF_3_, B(OCH_3_)_3_, B_2_O_3_, then perfluoro allyl fluorosulfate, CF_2_=CFCF_2_OSO_2_F (FAFS) is formed in modest to moderate yields (40%–60%) and >60% selectivity with respect to the corresponding sultone [2,3,4,5] according to the boron-mediated mechanism shown in Scheme 1.

Many different organic reactions can be carried out easily and with good yields with FAFS, namely (a) addition/elimination reactions with nucleophiles on the terminal allylic double bond by taking advantage both of FAFS’ FSO_3_^−^ anion being a very good leaving group, and the fact that attack by nucleophiles on sp^3^ carbon in highly fluorinated molecules does not occur [2]; (b) esterification on FAFS’ sulfur atom due to its elevated electronegativity and being F^−^ a good leaving group; (c) radical reactions, for example with hypofluorites such as CF_3_OF and FSO_2_CF_2_CF_2_OF [6], taking advantage of allylic resonance stabilization. Furthermore, Kostov and co-workers [7] have demonstrated that the allylic monomers generated from FAFS can be copolymerized with tetrafluoroethylene suggesting that FAFS’ derivatives can find useful applications in polymer chemistry.

The aim of the present work was to study various parameters concerning FAFS’ synthesis in order to increase yield and selectivity, to study the parameters that govern Addition/Elimination *vs.* Substitution at the sulfur atom, to synthesize and characterize a wide selection of fluoroallyl compounds for possible applications in polymer chemistry.

## 2. Results and Discussion

### 2.1. Synthesis of FAFS

Perfluoroallyl fluorosulfate (FAFS) has been known since 1981 [2] and its synthesis involves formally the insertion of SO_3_ in a C–F bond of hexafluoropropene (HFP) mediated by a boron catalyst [3,4] shown in Scheme 1. To date, the literature reports very little regarding both the synthesis and the utilization of FAFS as a source of perfluoroallyl-functionalities. In order to maximize FAFS’ yield and selectivity, we evaluated several parameters that might affect the outcome of the reaction:

- SO_3_/HFP molar ratio;
- Boron catalysts/SO_3_ molar ratio;
- SO_3_ concentration (oleum 20% (w/w) *vs.* oleum 65% (w/w) *vs.* 100% (distilled);
- Reaction temperature.

**Figure 1:**
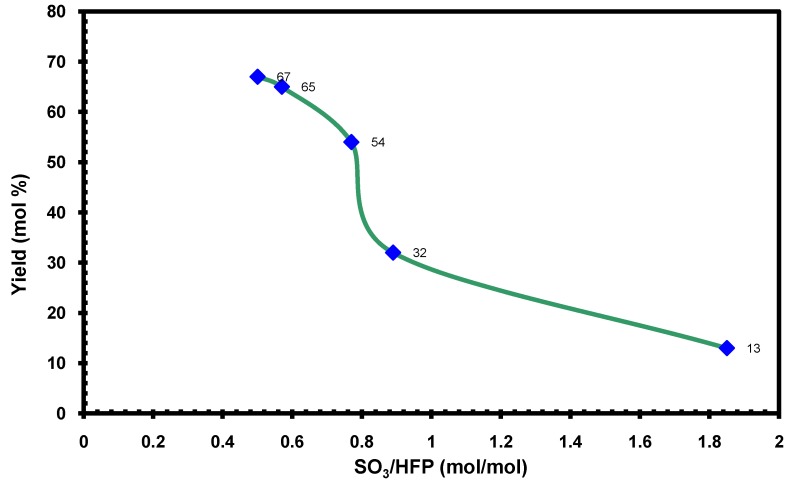
FAFS yield as a function of SO_3_/HFP molar ratio at 37 °C.

Figure 1 shows that there is a direct correlation between FAFS’ yield and the SO_3_/HFP ratio. The reaction temperature was always 37 °C. Surprisingly, the highest yields of FAFS are obtained at sub-stoichiometric ratios of SO_3_ with respect to the moles of HFP. The optimal molar SO_3_/HFP ratio was found to be 0.5:1. The explanation is that, as reported in the literature [8], monomeric SO_3_ tends to easily form dimers and trimers. Apparently, the dimerization and trimerization rate is faster than the rate of SO_3_ insertion in HFP. The SO_3_ dimers and trimers are not reactive with boron catalysts and tend to precipitate out of solution as inert solids thereby lowering FAFS’ yield. This effect is greatly enhanced when approaching a 2/1 SO_3_/HFP molar yield.

The boron-SO_3_ active catalyst complex shown in Scheme 1 can be achieved with several boron derivatives as shown in Table 1. The best results in terms of yield and selectivity are obtained by bubbling anhydrous BF_3_ in SO_3_ (100%) reaching a w/w BF_3_/SO_3_ ratio anywhere between 1.8 and 3.5 (Trial 4). In Trial 5 we tried to perform the synthesis with commercially available BF_3_*2 H_2_O simply because, being a solution at room temperature and pressure, it is easier to handle than anhydrous BF_3_ which is contained in a pressurized cylinder. The high HFP sultone selectivity suggests that BF_3_*2 H_2_O doesn’t form the boron-SO_3_ catalyst complex effectively. The same holds true for B(OCH_3_)_3_ (Trial 6). The only boron derivative that performed comparably to anhydrous BF_3_ was commercially available B_2_O_3_ (Trial 7) and can be considered a valid alternative to the more dangerous and difficult to handle anhydrous BF_3_.

Table 2 shows that the boron-SO_3_ catalyst complex doesn’t form in the presence of sulfuric acid (oleum at various SO_3_ concentrations) even at elevated BF_3_ w/w ratios *vs.* SO_3_. Unless pure, freshly distilled SO_3_ is employed, the principal reaction product will always be the sultone.

**Table 1:** FAFS selectivity and yield as a function of Boron catalyst type or born catalyst concentration.

Trial	Boron derivative	w/w *vs.* SO_3_ 100%	FAFS Selectivity (% mol)	PEP Sultone Sel. (% mol)	FAFS Yield (%)
1	anhydrous BF_3_	0	<5	>95	<5
2	anhydrous BF_3_	0.5–1	65	35	50
3	anhydrous BF_3_	1–1.6	85	15	55
4	anhydrous BF_3_	18.–3.5	95	<5	65
5	BF_3_*2H2O	1.03	48	52	48
6	B(OCH_3_)_3_	6	43	57	15
7	B_2_O_3_	4	80	20	45

**Table 2:** FAFS/HFP sultone molar selectivity as a function of [SO_3_].

Trial	SO_3_ type	Anhydrous BF_3_ (w/w *vs.* SO_3_)	FAFS/HFP sultone (mol/mol)
8	20% Oleum	3	1/99
		6	1/99
9	65% Oleum	3	4/96
		6	4/96
10	100% SO_3_	3	97/3
		6	95/5

Electrophilic ring opening of the HFP sultone described in the literature [9,10] will at most only give CF_3_CF=CFOSO_2_F and, following SO_3_ insertion at the terminal C–F bond, FSO_2_–O–CF=CFCF_2_–O–SO_2_F. Several attempts of such a ring opening were tried with no reaction even at high concentrations of BF_3_ and at reaction temperatures of 40–60 °C.

Early work by Krespan [3] demonstrated that FAFS can insert a second equivalent of SO_3_, obtaining FSO_2_OCF_2_CF=CFOSO_2_F, with the same mechanism as the first insertion of SO_3_ in HFP shown in Scheme 1. This side reaction contributes not only to lower FAFS selectivity, but also FAFS yield since it involves SO_3_ consumption. Data available from the literature [2,5] show that the reaction temperature for the boron catalyzed SO_3_ insertion in HFP was 50–150 °C and with rather low FAFS yields ranging from 20%–35%.

Table 3 shows the temperature dependence of FAFS’ selectivity employing the best reaction conditions found thus far: Use of freshly distilled SO_3_ (b.p. = 43 °C), SO_3_/HFP molar ratio = 0.5/1, anhydrous BF_3_ with BF_3_/SO_3_ w/w % =1.8. Along with the optimal reaction conditions just mentioned, Table 3 shows that the best temperature for this reaction is <40 °C.

**Table 3:** FAFS and its side reaction products as a function of reaction temperature.

Trial	T (°C)	FAFS/FSO_2_–O–CF_2_CF=CFO–SO_2_F/Sultone
11	37	95/4/1
12	60	35/60/5
13	100	20/75/5

### 2.2. FAFS Regiochemistry

FAFS is an asymmetrical olefin and therefore it will have two centers of attack about the CF_2_=CF– bond: The C-3 terminal olefin carbon or the C-2 internal carbon. Furthermore, FAFS also embodies two distinct electrophilic centers: The terminal olefin and the electrophilic sulfur atom as well. These electronic features give FAFS a variety of different regiochemistries depending on the nature of the reaction.

#### 2.2.1. Radical reactions

**Scheme 3:**
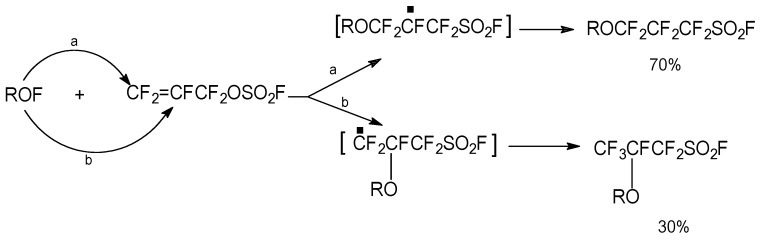
FAFS regioselectivity with radicals employing a general hypofluorite ROF.

As with all asymmetrical olefins [11] the attacking radical will add to the carbon center that will generate the most stable radical intermediate, which in this case is the terminal C-3 carbon center. The radical sum of a general hypofluorite ROF gave the product distributions and reaction mechanisms shown in Scheme 3.

The different molar product distribution reflects the relative stability of a primary *vs.* secondary radical on a fluorinated carbon. The following hypofluorites were added to FAFS with moderate to good yields: CF_3_OF, FSO_2_CF_2_CF_2_OF and CF_2_(OF)_2_.

#### 2.2.2. Nucleophilic reactions

It will be shown that FAFS, due to its electrophilic nature, is quite reactive towards a number of different nucleophiles, including for example alcohols, yielding the corresponding fluorinated allyl ethers. Unlike what was previously reported in the literature [4], it is subject to nucleophilic substitution by alcohols both without basic catalysis (*i.e.*, directly with the protonated alcohol) as well as with the corresponding conjugate base. Employing an excess of an alcohol in the presence of FAFS one always obtains the corresponding allyl ether. Table 4 shows the selectivities and product distributions of some typical hydrogenated and partially fluorinated alcohols both with (Na^+^ as the cation) and without basic catalysis.

Of course, with the base catalyzed nucleophilic addition to FAFS, one must employ stoichiometric quantities of the alcohol in order to avoid a second addition of the alcoholate to the allyl ether yielding, from a general alcohol ROH, RO–CF_2_CFHCF_2_–OR. The proton in the fluorinated propyl chain comes from the solvent (generally CH_3_CN or glymes) employed.

**Table 4:** FAFS regioselectivity with several oxygen nucleophiles.

Trial	Nucleophile	Conv. (FAFS)	Products (selectivity %)
14	CH_3_OH	100%	CH_3_OCF_2_CF=CF_2_ (100%)
15	CH_3_ONa	100%	CH_3_OCF_2_CF=CF_2_ (100%)
16	C_6_H_5_OH	54%	C_6_H_5_OCF_2_CF=CF_2_ (86%)/CF_2_=CFCF_2_OSO_2_OC_6_H_5_ (14%)
17	C_6_H_5_ONa	98%	C_6_H_5_OCF_2_CF=CF_2_ (87%)/CF_2_=CFCF_2_OSO_2_OC_6_H_5_ (13%)
18	CF_3_CH_2_OH	46%	CF_3_CH_2_OCF_2_CF=CF_2_ (85%)/CF_2_=CFCF_2_OSO_2_OCH_2_CF_3_ (15%)
19	CF_3_CH_2_ONa	97%	CF_3_CH_2_OCF_2_CF=CF_2_ (86%)/CF_2_=CFCF_2_OSO_2_OCH_2_CF_3_ (14%)
20	C_6_H_5_CH_2_OH	96%	C_6_H_5_CH_2_OCF_2_CF=CF_2_ (95%)/CF_2_=CFCF_2_OSO_2_OCH_2_C_6_H_5_ (5%)
21	C_6_H_5_CH_2_ONa	99%	C_6_H_5_CH_2_OCF_2_CF=CF_2_ (94%)/CF_2_=CFCF_2_OSO_2_OCH_2_C_6_H_5_ (6%)
22	C_6_F_5_OH	30%	C_6_F_5_OCF_2_CF=CF_2_ (86%)/CF_2_=CFCF_2_OSO_2_OC_6_F_5_ (14%)
23	C_6_F_5_ONa	100%	C_6_F_5_OCF_2_CF=CF_2_ (90%)/CF_2_=CFCF_2_OSO_2_OC_6_F_5_ (10%)

Table 4 shows that there are at least two distinct regiochemistries involved in the nucleophilic addition to FAFS: One yields an allyl ether (main product) and the other a sulfate ester (minor product).

**Scheme 4:**
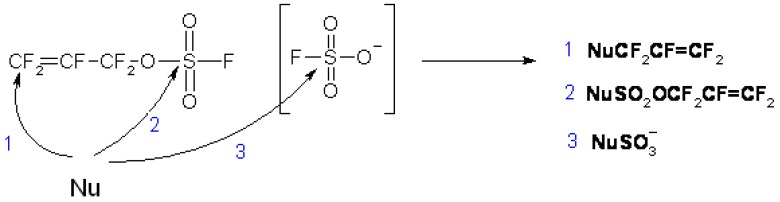
Different modes of nucleophilic attack of a general nucleophile Nu: on FAFS.

Scheme 4 shows the three possible sites of attack of a general nucleophile to FAFS. Taking reaction 1 depicted in Scheme 4 into consideration, unlike the hypofluorite radical addition shown in Scheme 3, nucleophilic attack was almost exclusively (>98.5/1.5) observed on the terminal olefin yielding a secondary anion. Pathway 1 is an Addition/Elimination (A/E) mechanism of the nucleophile to FAFS’ terminal double bond. The main driving force of the reaction is the powerful leaving group FSO_3_^−^. Furthermore, it is known from the literature that attack by a nucleophile on the sp^3^ carbon in highly fluorinated molecules does not occur [2]. On the other hand, Pathway 2 is a Substitution (S_N_) reaction by the nucleophile on FAFS’ sulfur atom yielding a sulfate ester. The driving force of this reaction is the electropositive sulfur and the relatively good leaving group, F^−^. In very few instances and with particularly acidic fluoro alcohols, Pathway 3 was also observed: Once FSO_3_M (M = H, Metal) is formed by Pathway 1, a second nucleophile can attack FSO_3_M’s electropositive sulfur atom, displace F^−^ and form the general product NuOSO_2_M.

As can be seen from Table 5 there is a direct correlation between the alcohol’s pK_a_ and the A/E *vs.* S_N_ product distribution shown in Table 4.

**Table 5:** Correlation between an oxygen nucleophile’s pK_a_ and substitution selectivity on FAFS.

ROH	pK_a_	CF_2_=CFCF_2_OSO_2_OR %
C_6_H_5_OH	9,9	14
CF_3_CH_2_OH	12,4	15
C_6_H_5_CH_2_OH	15	5
CH_3_OH	16	0

Alcohols with a pK_a_ less than 13 and therefore relatively “acidic” either by resonance effect (as in phenol, pK_a_ = 9.9) or by inductive effect (as in trifluoroethanol, pK_a_ = 12.4) give a higher percentage of S_N_ product (Pathway 2). On the other hand, methanol (pK_a_ = 16) and benzyl alcohol (pK_a_ = 15), which are more basic, give almost exclusively the A/E product (Pathway 1).

Another interesting feature that emerges from the data presented in Table 4 is that the A/E *vs.* S_N_ selectivity remains practically unchanged regardless to whether the nucleophile is a charged species (oxyanion) or a species with a free unpaired electron doublet on the oxygen atom (alcohol). This leads us to assert that the regioselectivity observed is determined not only by the particular electronic nature of the nucleophile (pK_a_ due to resonance or inductive effects, oxyanion *vs.* protonated alcohol) but also on the electronic nature of FAFS’s terminal olefin *vs.* FAFS’s sulfur atom.

**Figure 2:**
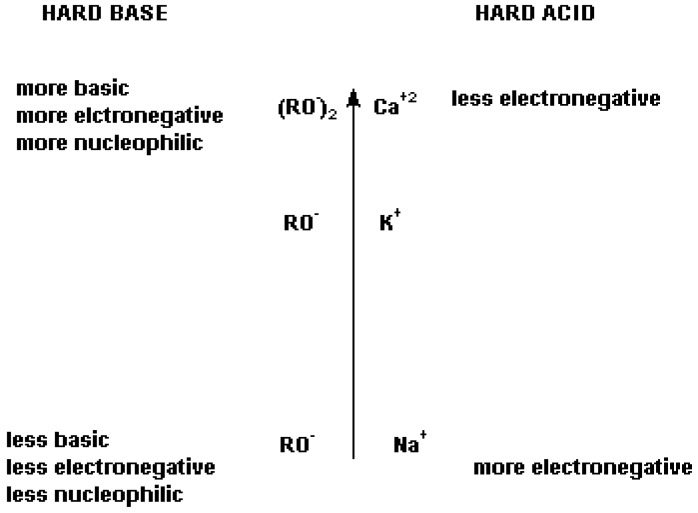
Lewis [12] and Pearson’s [13] representation of the “Hard-Soft-Acid-Base theory concerning anions and cations.

Finally, regardless of the regiochemistry observed, the base catalyzed addition of an alcohol to FAFS is a much faster reaction as evidenced by the higher conversions of FAFS with the conjugate base *vs.* the free alcohol. The striking differences in regioselectivities observed thus far, led us to investigate if there was a “cation” effect on regioselectivity as well. It is known in the literature that the electronic nature of the nucleophiles is not only governed by inductive and mesomeric effects, but also by the Hard-Soft-Acid-Base theory of Lewis [12] and Pearson [13] whose trends are shown in Figure 2.

It becomes clear that, based on the regiochemistry considerations made thus far, varying the nucleophile’s cation may vary the regiochemistry for the nucleophilic attack on FAFS.

**Figure 3:**
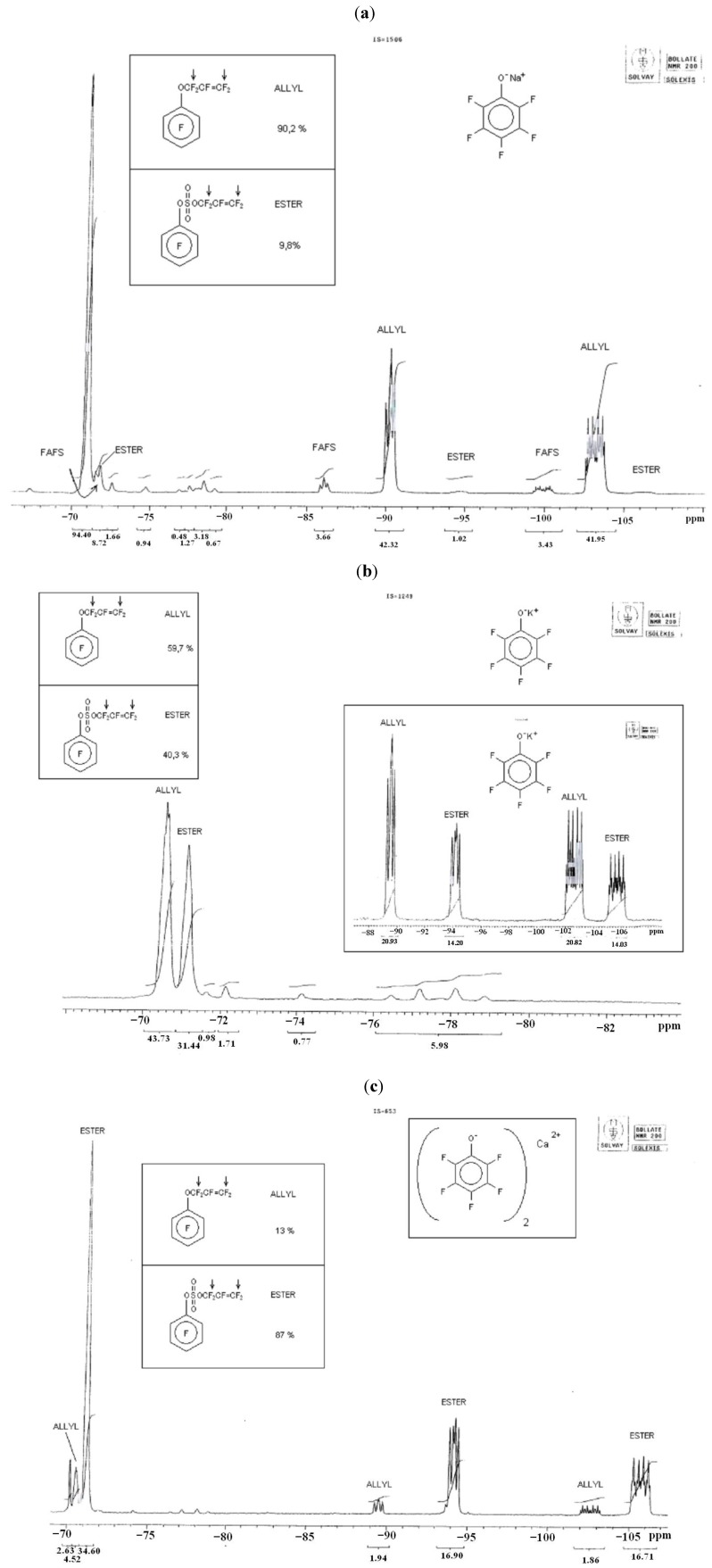
^19^F-NMR (200 MHz) spectrum of the Addition/Elimination (“Allyl”) *vs.* Substitution (“Ester”) products after reaction between FAFS and (**a**) C_6_F_5_ONa, (**b**) C_6_F_5_OK and (**c**) (C_6_F_5_O)_2_Ca.

We therefore used pentafluorophenol (pK_a_ = 8.9) [14] as a model compound to study the effects of Ca^2+^, K^+^ and Na^+^ cations on regiochemistry. The averaged results of the cation effect on regiochemistry are shown in Table 6 along with the ^19^F-NMR details shown in Figure 3a–c. All reactions were performed in anhydrous THF with a stoichiometric quantity of nucleophiles with respect to the moles of FAFS.

**Table 6:** Addition/Elimination *vs.* Substitution molar selectivities on FAFS as a function of the cation.

Trial	Nucleophile	T_R_(°C)	C_6_F_5_OCF_2_CF=CF_2_/C_6_F_5_O–SO_2_–OCF_2_CF=CF_2_
24	(C_6_F_5_O)_2_Ca	40 °C	13/87
25	C_6_F_5_OK	40 °C	60/40
26	C_6_F_5_ONa	40 °C	90/10

The experimental data reported in Table 6 confirm the HSAB theory summarized in Figure 3: In going from a Na^+^ cation to a Ca^2+^ cation the hard base alcoholate becomes progressively more ionically charged or, in other words, less covalently bound, and therefore more susceptible to attacking FAFS’ very electropositive sulfur atom. Therefore, the main product of the nucleophilic addition of sodium perfluoro phenolate and FAFS is C_6_F_5_OCF_2_CF=CF_2_ (A/E selectivity = 90%), the minor product is CF_2_=CFCF_2_OSO_3_C_6_F_5_ (S_N_ selectivity = 10%); on the other hand, the main product employing calcium phenolate is the sulfate ester (S_N_ selectivity = 87%), and the minor product is the corresponding perfluoro allyl ether (A/E; selectivity= 13%). Pure compounds from Trials 24 and 26 were isolated by flash silica gel chromatography and identified by GC-MS. This permitted us to unequivocally assign the ^19^F-NMR frequencies (in ppm) observed in Figure 3a–c and shown in Table 7.

**Table 7:** Specific ^19^F-NMR (300 MHz) frequencies observed for Addition/Elimination (a–c’)*vs.* Substitution (d–f’) products.

	a	b	c	c’	d	e	f	f’
–O^a^CF_2_^b^CF=^c,c’^CF_2_	−70.6	−189	−89.8	−103.2	-	-	-	-
–OSO_2_O-^d^CF_2_^e^CF=^f,f’^CF_2_	-	-	-	-	−71.2	−189.5	−94.2	−105.8

Therefore, in a base catalyzed addition between an alcohol’s conjugate base and FAFS, in order to selectively obtain an A/E product, *i.e.*, an allyl ether as the main product, the cation must be Na^+^.

**Scheme 5:**
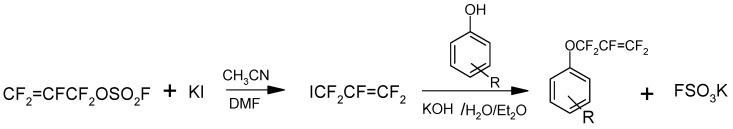
Allyl iodide mediated synthesis of allyl ethers.

One of the few well documented A/E reactions in the literature is the sum of a metal halide MX, to FAFS where X = I, Br, Cl [4]. It becomes immediately obvious that ICF_2_CF=CF_2_ is a hydrolytically stable synthon of FAFS, but with only one possible regioisomer obtainable due to the absence of the electrophilic sulfur atom. Therefore, if quantitative selectivity towards A/E is necessary, ICF_2_CF=CF_2_ can be synthesized *in situ* (see Experimental), according to a slightly modified reaction procedure with respect to the literature [4], and immediately added to the nucleophile according to Scheme 5. Complete regioselectivity towards the allyl ether is obtained with isolated yields ranging from 55%–85% depending upon the alcohol.

### 2.3. Addition/Elimination Reactions with FAFS

**Scheme 6:**
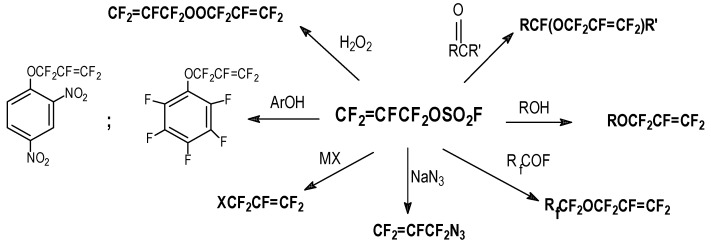
Generalized product library possible with FAFS.

FAFS is a very versatile monomer and can be employed in a wide variety of nucleophilic reactions obtaining, according to the rules and mechanisms just discussed, a plethora of allylic derivatives. Scheme 6 summarizes some of these derivatives in a general manner.

#### 2.3.1. Aromatic and aliphatic alcohols

Table 8 summarizes some specific examples of A/E of aliphatic and aromatic alcohols, both hydrogenated and partially fluorinated. As can be observed, with the exception of methanol and benzyl alcohol, all other alcohols and phenols have a pK_a_ < 13 and therefore need a basic catalysis and Na^+^ as the counter cation in order to have both good conversions of FAFS and especially a high selectivity towards A/E, as previously described.

**Table 8:** Reaction selectivities and Addition/Elimination yields for the addition of several aliphatic and aromatic alcohols with different pK_a_ to FAFS.

ROH	pK_a_	Sel. (%)	A/E Isolated Yield
CH_3_OH	16	99%	80%
C_6_H_5_CH_2_OH	15	95%	50%
CF_3_CH_2_ONa	12.4	85%	65%
C_6_H_5_ONa	9.9	86%	87%
3-CF_3_-Ph-ONa	9.5	92%	48%
C_6_F_5_ONa	8.9	90%	60%
4-NO_2_-Ph-ONa	7.2	90%	75%
2,4-NO_2_-Ph-ONa	4	85%	55%

We observed that the best solvents for all of the reactions were aprotic ones such as anhydrous CH_3_CN or THF. In these solvents FSO_3_Na, the elimination product, is practically insoluble; this physical-chemical condition helps push the reaction to the right favoring high FAFS conversions and minimizing the side reaction of Pathway 3, shown in Scheme 4. In some instances diglyme proved to be a good solvent due to its excellent solvation properties. Care must be taken if employing diglyme: If the reaction pH drops, FSO_3_H is a strong enough acid to protonate diglyme yielding inverse Williamson [15] degradation products which react with FAFS, lowering the reaction yield. The isolated yield of the allyl ethers shown varies depending on the specific substrate.

**Scheme 7:**
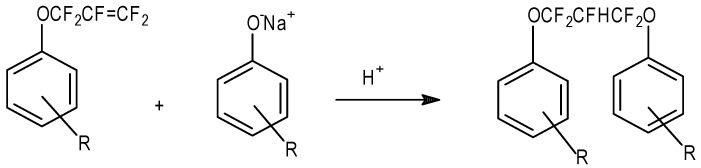
Main side reaction product if excess nucleophile is employed in the in the base-promoted addition of an alcohol to FAFS.

As shown in Table 8 most of the Nucleophilic A/E reactions were performed with basic catalysis; it was therefore preferable to operate in substoichiometric amounts of the nucleophiles in order to avoid the side reaction shown in Scheme 7.

For this reason diglyme was often chosen as the solvent since it effectively solubilizes many sodium conjugate bases of alcohols. In this way it is possible to add the dissolved nucleophile to FAFS keeping it in molar defect with respect to the allyl ether reaction product. The necessary proton for protonation of the intermediate fluorinated carbanion can come from traces of H_2_O or the solvent itself. Electron withdrawing substituents on the aromatic ring such as F–, CF_3_–, NO_2_– simply contribute to lowering the pKa of the phenol, but have no noticeable effects on A/E *vs.* S_N_ selectivity. The rules that govern the regioselectivity of a nucleophile described in the previous section are therefore obeyed.

#### 2.3.2. Acyl fluorides

**Scheme 8:**
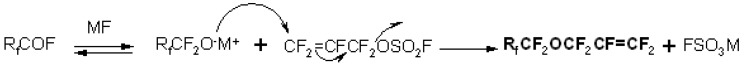
Generalized reaction scheme for the addition of a fluorinated acyl fluoride to FAFS.

Acyl fluorides having the general formula R_f_COF, placed with a stoichiometric amount of a metal fluoride MF (M = Na, K, Cs), react with FAFS yielding a perfluoroallyl alkyl ether as shown in Scheme 8.

R_f_ may be either F^−^ or a perfluorinated alkyl chain of any length. Perfluoroallyl alkyl ethers have already been synthesized by Krespan [16] and employed in polymerization reactions with fluorinated olefins [17,18]. The reported literature yields were rather low; we therefore evaluated parameters such as reaction temperature, solvent and reaction pressure in order to try to improve Krespan’s yields and selectivities.

The rate-determining step is the acyl fluoride <==> alcoholate equilibrium. The alcoholate, due to the inductive effect of –CF_2_– α to the oxyanion is less nucleophilic than its hydrogenated counterpart. It is furthermore known in the literature [19] that the equilibrium reaction in Scheme 8 is shifted to the right with increasing reaction temperature.

Using CF_3_COF as a model acyl fluoride in the presence of anhydrous KF we found that the maximum concentration of alcoholate, CF_3_CF_2_O^−^K^+^, was 70% obtained at 30 °C as determined by ^19^F-NMR (+22 ppm, sharp, for –COF *vs.* −18 ppm, broad, for –CF_2_O^−^ with CFCl_3_ as an internal standard). Unfortunately, performing the reaction with FAFS at this temperature yielded only CF_3_CF=CF_2_ (HFP), SO_2_F_2_, FSO_3_^−^ and CF_2_=CFCOF (ACF, traces). No perfluoroallyl ethers were detected.

**Scheme 9:**
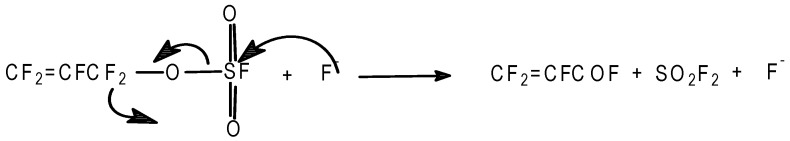
Desulfurilation of FAFS by F^(−)^—Reaction products.

The presence of ACF and SO_2_F_2_ indicated that there must have been a nucleophilic attack by F^−^ anion on FAFS’ sulfur atom; this reaction is shown in Scheme 9. The literature reports that catalytic desulfurilation reactions such as this one generally occur at high reaction temperatures (>100 °C) but it is plausible that it may also occur at much lower temperature with very reactive compounds such as FAFS.

**Scheme 10:**
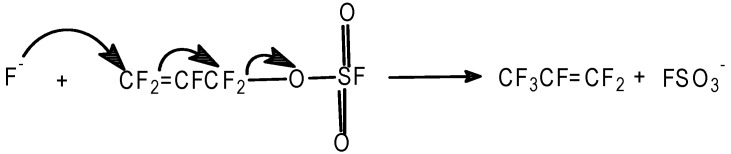
Addition/Elimination by F^(−)^ on FAFS—Reaction products.

HFP and FSO_3_^−^ are generated by A/E of F^−^ on FAFS as shown in Scheme 10.

Since F^−^ anions are always present in the reaction medium due to the equilibrium shown in Scheme 8, increasing the reaction temperature will effectively shift the equilibrium to the right, but it will at the same time favor the side reactions just described. We therefore attempted the addition of an acyl fluoride to FAFS at much lower temperatures. Table 9 shows the results obtained.

**Table 9:** Low-temperature (−20 °C~r.t) addition of different fluorinated acyl fluorides to FAFS—Yields and selectivities.

Trial	R_f_COF	Perfluoroalkyl Allyl Ether	Yield (Selectivity)	b.p. (°C)
27	COF_2_	CF_3_OCF_2_CF=CF_2_	54(85)	11–12
28	CF_3_COF	CF_3_CF_2_OCF_2_CF=CF_2_	86(96)	39–40
29	CF_3_CF_2_COF	CF_3_CF_2_CF_2_OCF_2_CF=CF_2_	67(99)	48–49
30	FSO_2_CF_2_COF	FSO_2_CF_2_CF_2_OCF_2_CF=CF_2_	84(94)	105

Depending on the acyl fluoride employed (see Experimental), the reaction temperatures varied from −20 °C to r.t., in these conditions the acyl fluoride <==> alcoholate equilibrium is shifted to the left but, unlike F^−^ anions, the alcoholate slowly reacts with FAFS therefore obtaining good yields and selectivities with minimal formation of byproducts. Table 10 shows the yields and selectivities obtained by varying the solvent, reaction temperature and metal fluoride for the synthesis of CF_3_OCF_2_CF=CF_2_.

Aprotic solvents favor the A/E reaction of F^−^ anion on FAFS yielding HFP and FSO_3_^−^ while the absence of solvents favors the catalytic desulfurilation. The most favorable reaction conditions were those of Trial 27e and they were applied to all of the other acyl fluorides reported in Table 9.

**Table 10:** Addition of COF_2_ to FAFS—Yields, selectivities and side-reaction products as a function of reaction temperature and solvent (ACF = Acryloyl fluoride, CF_2_=CFC(=O)F).

Trial	MF	Solvent	T_R_	Yield (%) (Selectivity %)	By-Products
27a	CsF	Tetraglyme	150 °C	10 (30)	HFP
27b	CsF/NaF	-	RT	/ (85)	SO_2_F_2_/ ACF
27c	CsF/NaF	-	100 °C	/	SO_2_F_2_/ ACF
27d	KF	Diglyme	−20 °C	9 (98)	HFP
27e	KF	Diglyme	−5 °C	54 (85)	HFP

#### 2.3.3. Halides

Table 11 shows the perfluoroallyl halides obtained by the A/E reaction with KI, KBr and KCl. Based on the reaction temperature necessary for complete conversion of FAFS the following reactivity scale was established: I^−^ >> Br^−^ > Cl^−^.

**Table 11:** Allyl halide yield as a function of the halide.

Nucleophile	T_R_ (°C)	t_R_ (h)	FAFS Conversion	XCF_2_CF=CF_2_ Yield
KI	3 °C	2.5	100%	85%
KBr	20 °C	2.5	100%	56%
KCl	50 °C	2.5	98%	31%

All reactions were carried out for 2.5 h and the solvent system was 0.98 CH_3_CN/0.02 DMF (w/w). Changing the solvent to diglyme, which is known to solubilize inorganic salts well, didn’t appreciably change the conversion times or the yields obtained. We found that when CH_3_CN was employed, a very low percentage of DMF was necessary to help solubilize the metal halide. All three perfluoroallyl halides are synthons of FAFS and react in the same way FAFS does. ICF_2_CF=CF_2_, being the most easily synthesized perfluoroallyl halide, was obtained *in situ* when it was absolutely necessary not to have A/E *vs.* S_N_ competition. Furthermore, unlike FAFS, ICF_2_CF=CF_2_ is hydrolytically stable at least up to r.t. and can be employed in those nucleophilic reactions where an anhydrous solvent is not available.

#### 2.3.4. Azides

**Scheme 11:**
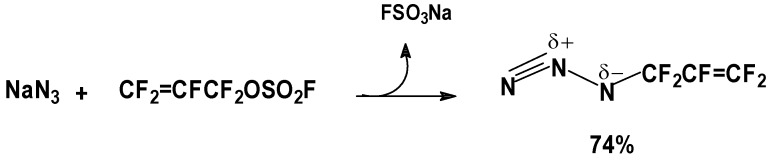
Allyl azide synthesis.

Reacting FAFS in an anhydrous CH_3_CN/NaN_3_ slurry at r.t. for 3 h the following main product shown in Scheme 11 has been identified by ^19^F-NMR.

#### 2.3.5. H_2_O_2_

**Scheme 12:**
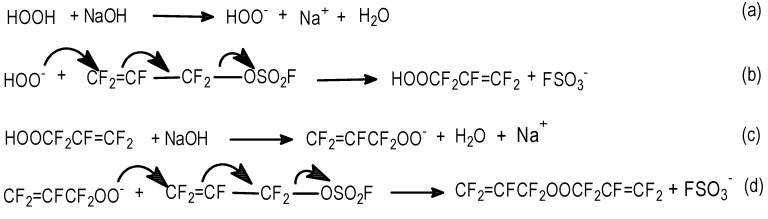
Diallylperoxide reaction mechanism.

The nucleophilic A/E sum of H_2_O_2_ to FAFS was studied both in an aqueous biphasic system [21] as well as in an anhydrous system. Scheme 12 shows the reactions involved in the peroxidation reaction.

##### 3.3.5.1. Aqueous conditions

The reaction was carried out employing commercial aqueous 30% H_2_O_2_ (w/w; 0.5–5 equiv.) in the presence of an inert fluorinated solvent (CFC 113, C_6_F_14_, CF_3_OCFClCF_2_Cl– “Methyl Adduct”–; solvent/30% H_2_O_2_ = 4:1 by volume) and NaOH (1–2 equiv. *vs.* H_2_O_2_) between 0–20 °C for a total reaction time of 10 min as already described elsewhere for similar reactions [21]. Table 12 shows the results obtained.

**Table 12:** H_2_O_2_ addition to FAFS in aqueous conditions–Products and selectivities as a function of Solvent/H_2_O ratio (v/v).

Trial	H_2_O_2_ (eq.)	Solvent/H_2_O (v/v)	T_R_ (°C)	t_R_ (min)	FAFS Conversion	Products (Selectivity)
31	1	5/1	0	10	0%	-
32	1	1/1	0	10	10%	CF2=CFCF2OOCF2CF=CF2 (89%)
33	5	0	0–8	9	100%	CF2=CFCF2OOCF2CF=CF2 (26.7%)
						(CF2=CFCO2)2 (11.8%)
						(HOOCCFHCO2)2 (3.5%)
						(CF3CFHCO2)2 (3.2%)
						Other acids + peracids (54.9%)

Trials 31 and 32 show that one major problem of the reaction is the contact between FAFS and H_2_O_2_ in the heterogeneous system, which doesn’t allow high conversions of FAFS. In trial 33 the fluorinated solvent (CFC 113) was omitted in the attempt to create a better contact between the reagents. At the end of the reaction phase separation was not clear cut suggesting the presence of fluorinated acids and peracids which act as surfactants. Nonetheless, at 100% FAFS conversion, the desired perfluoroallyl alkyl peroxide was obtained with 26.7% selectivity along with numerous other peroxidic compounds shown in Table 13 where we also report the concentration of each peroxide as a function of time, at 20 °C, as determined by quantitative ^19^F-NMR.

During the kinetic measurements shown in Table 13, ^19^F-NMR analyses indicated that the organic material decomposed significantly to inorganic fluorides, (mainly MF and FSO_3_^−^) and gaseous byproducts identified as CF_2_=CFCF=CF_2_ (PFBD) and CO_2_. Table 14 shows the progress of the % molar decomposition at 20 °C as function of time.

**Table 13:** Sum of aqueous H_2_O_2_ to FAFS—Products observed (^19^F-NMR) and their decomposition as a function of time at 20 °C.

Compound	[c] (M) at 0.167 h	[c] (M) at 18 h	[c] (M) at 24 h	[c] (M) at 36 h
1: CF_2_=CFCF_2_OOCF_2_CF=CF_2_	0.312	0.0319	0.0289	0.0101
2: CF_2_=CFCOF	0	0	0	0
3: CF_2_=CFCOOH	0.264	0.01135	0.01579	0.00316
4: CF_2_=CFC(=O)OOC(=O)CF=CF_2_	0.1381	0.000468	0	0
5: HOOCCFHCOOH	0.2516	0.0445	0.0498	0.02282
6: HOOCCFHC(=O)OOC(=O)CFHCOOH	0.04118	0.000585	0.000222	0
7: CF_3_CFHCOOH	0.1264	0.02094	0.03194	0.00878
8: CF_3_CFHC(=O)OOC(=O)CFHCF_3_	0.0373	0.000269	0	0
9: CF_2_=CFC(=O)OOH	0	0.0819	0.02094	0.02691
10: CF_3_CFHC(=O)OOH	0	0.09594	0	0.06154
11: HOOCCFHC(=O)OOH	0	0.1041	0.1802	0.0875

The decomposition observed in Table 14 is to be attributed not only to the individual thermal k_d_ of the peroxides but also to the presence of H_2_O due to poor phase separation of the aqueous and organic phases at the end of the reaction. It is known that hydrolytic decompositions, especially for fluorinated diacyl peroxides, is several orders of magnitude faster than the thermal decomposition rate [21].

**Table 14:** Sum of aqueous H_2_O_2_ to FAFS—Decomposition of all of the organic products as a function of time at 20 °C.

	t = 10 min	t = 18 h	t = 24 h	t = 36 h
% decomposition	0%	62%	65%	76%
% residual organics	100%	33,2%	28,3	18,9%

**Scheme 13:**
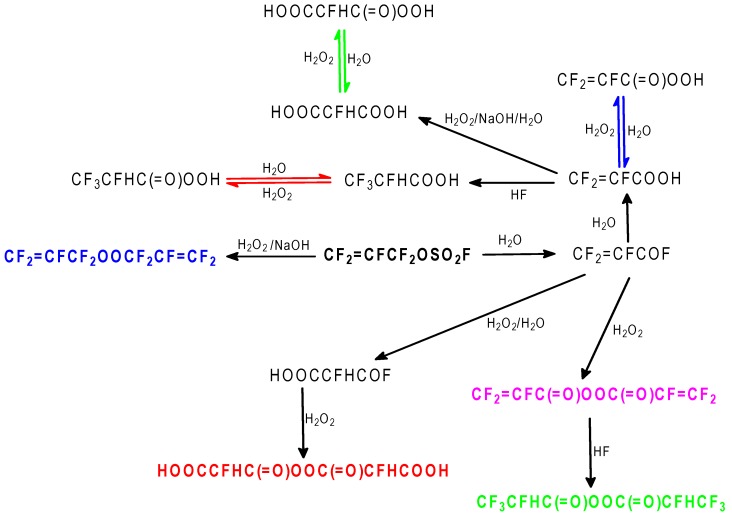
Sum of aqueous H_2_O_2_ to FAFS—Reaction pathways that lead to the observed reaction products.

**Scheme 14:**
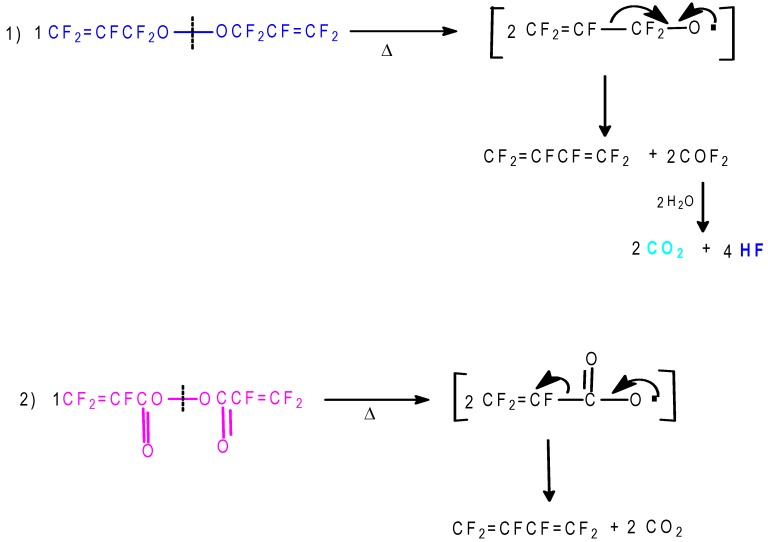
Sum of aqueous H_2_O_2_ to FAFS—Thermal (20 °C) decomposition products of dialkyl- and diacil peroxides.

Scheme 13 and Scheme 14 show the reactions involved that justify all of the peroxidic species identified in Table 13. The thermal decomposition rate constants at 20 °C, k_d_ and the respective half-lives of peroxides **1**, **4**, **6** and **8** of Table 13 were calculated according to a first order radical decomposition mechanism [21,22,23,24] defined by Equations 1 and 2:

ln[Peroxyde]_t_ = −k_d_t + ln[Peroxyde]_o_ (1)

t_1/2_ = ln2/k_d_ (2)

**Figure 4:**
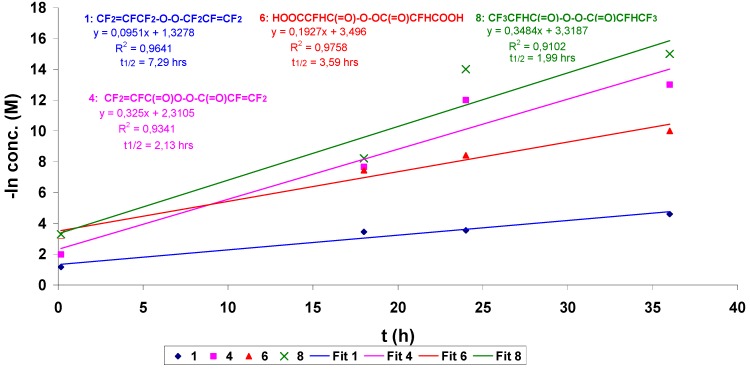
Sum of aqueous H_2_O_2_ to FAFS—linear regression for the determination of the thermal decomposition rate constant k_d_ and half-life t_1/2_ at 20 °C for the peroxides observed.

Figure 4 and Table 15 show respectively the decomposition kinetics and the linear regression obtained from the data of Table 12 and used to determine both k_d_ and t_1/2_ for peroxides **1**, **4**, **6** and **8**.

**Table 15:** Sum of aqueous H_2_O_2_ to FAFS—k_d_ and t_1/2_ at 20 °C for the peroxides observed.

Peroxide	k_d_ × 10^8^ (s^−1^)	t_1/2_ (h)
1. CF_2_=CFCF_2_OOCF_2_CF=CF_2_	2642	7,29
4. (CF_2_=CFCO_2_)_2_	9028	2,13
6. (HOOCCFHCO_2_)_2_	5353	3,59
8. (CF_3_CFHCO_2_)_2_	9678	1,99

We can observe in Table 15 that the perfluoroallyl peroxide **1** has a smaller k_d_ and a longer t_1**/**2_ compared to the other peroxides. The k_d_ of the fluorinated diacyl peroxides **4**, **6** and **8** can’t be compared with those of other diacyl peroxides found in the literature [21,22,23] since their structures and MW are too different from those cited. It is in fact known that there is a good correlation between diacyl peroxide structure and MW with the stability of the radical [22,24] coming from the homolytic cleavage of the diacyl peroxide –O–O– bond. Instead, comparing the peroxides of Table 14 we can say that the CF_2_=CFCF_2_O• radicals obtained from the homolytic cleavage of the –O–O– dialkyl peroxide **1** bond are less stable than the R_f_C(=O)O• radicals from homolytic cleavage of the –O–O– diacyl peroxide bonds of peroxides **4**, **6** and **8** (longer t_1/2_ and a smaller k_d_).

**Figure 5:**
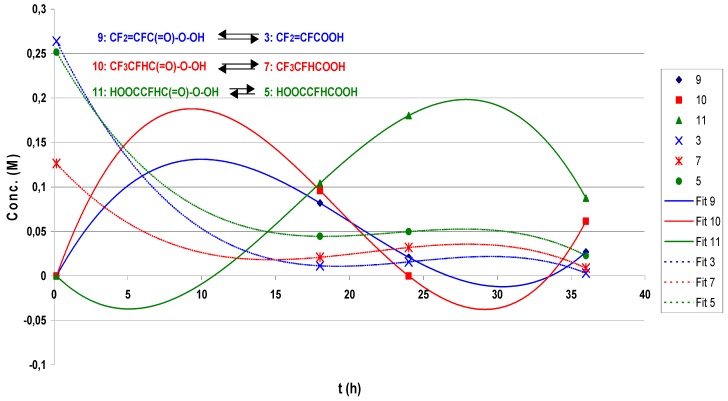
Sum of aqueous H_2_O_2_ to FAFS—Acid-peracid equilibria.

The correlation of the molar concentrations of the carboxylic acids **3**, **7**, and **5** and the respective peracids **9**, **10** and **11** as a function of time is reported in Figure 5 (data from quantitative ^19^F-NMR). The curves in Figure 5 were obtained by fitting the experimental concentrations reported in Table 13 to a 3rd degree polynomial equation. The acid-peracid couples (acids: Dotted curves; peracids: Whole curves) are essentially complementary: As the concentration of a peracid increases, the corresponding acid concentration decreases.

##### 2.3.5.2. Anhydrous conditions

The presence of water in the FAFS peroxidation gives several compounds having a peroxidic bond.

In order to increase the desired perfluorodiallyl peroxide **1** selectivity and decrease the total number of acids and peracids, we tested three different anhydrous or nearly anhydrous reactions with H_2_O_2_ and FAFS:

- *Method A*
  Na_2_O_2_ + H_2_SO_4_(96%) ---------> H_2_O_2_(96% +H_2_O 4%) + Na_2_SO_4_ ---------> (CF_2_=CFCF_2_O)_2_
  THF; 0 °C FAFS; CFC 113.
- *Method B*
  Na_2_O_2_ + 2 H_2_O_2_(30%) ------> H_2_O_2_(30%) + 2 NaOH +O_2_ ------> (CF_2_=CFCF_2_O)_2_
  CH_2_Cl_2_; 0 °C FAFS.
- *Method C*
  H_2_O_2_(30%) + CaH_2_ ------> H_2_O_2_(100%) + Ca(OH)_2_ + 2H_2_ -------> (CF_2_=CFCF_2_O)_2_
  CH_3_CN; N_2_; −15 °C FAFS; CH_3_CN.

The results are summarized in Table 16. Method A which involved nearly anhydrous and acidic conditions gave no reaction and FAFS was recovered completely. Method B had approximately the same molar content of H_2_O as Method A, but with a basic pH. In this case FAFS converts completely and yields five products (as compared to 11 different products in the aqueous reaction conditions): The desired perfluorodiallyl peroxide has a selectivity = 32%. Method C is completely anhydrous and yields almost exclusively perfluorodiallyl peroxide **1**. The drawback of this method, is that it generates 100% H_2_O_2_, which is potentially explosive. The data presented in this section suggest that the selectivity of CF_2_=CFCF_2_–O–O–CF_2_CF=CF_2_ depends greatly on the anhydrousness of the reaction.

**Table 16:** Addition of anhydrous H_2_O_2_ to FAFS—Product distributions and selectivities.

Trial	Method	FAFS Conversion (%mol)	Products (Selectivity %)
34	A	0%	No Reaction
35	B	100%	CF_2_=CFCF_2_O-O-CF_2_CF=CF_2_ (32%)
			(CF_3_CFHCO_2_)_2_ (2,2%)
			HOOCCFHCOOH (17,3%)
			(HOOCCFHCO_2_)_2_ (42,8%)
			CF_3_CFHCOOH (5,8%)
36	C	90%	CF_2_=CFCF_2_O-O-CF_2_CF=CF_2_

#### 2.3.6. Ketones

**Scheme 15:**
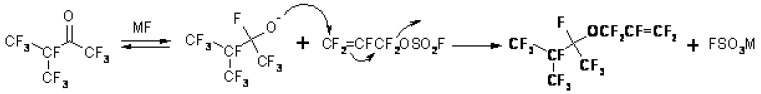
Reaction mechanism for the addition of a ketone to FAFS.

Scheme 15 shows the synthesis of a branched allyl ether that can be obtained by reacting a ketone, in this specific case perfluoro isopropyl trifluorometyl ketone, with a metal fluoride followed by addition of FAFS to the alcoholate in much the same manner as was done with the addition of perfluorinated acyl fluorides to FAFS in section (*ii*). The perfluroketone is easily prepared by reacting a perfluorinated olefin, in this case HFP, with a stoichiometric amount of a fluorinated acyl fluoride, in this case acetyl fluoride, in the presence of a catalytic amount of a metal fluoride.

As with the previously discussed acyl fluorides, the alcoholate is formed in the presence of an aprotic solvent, such as anhydrous diglyme which solvates well the oxyanion thereby shifting the equilibrium reaction to the right much like Trial 27e in Table 10, at reaction temperature ranging between 0–5 °C. The only major difference encountered in the reaction of the branched fluorinated alcoholate of Scheme 15 and the linear fluorinated alcoholates of Table 9 is the reaction time: branched alcoholates reacted with FAFS much more slowly (10–12 h) than linear alcoholates (3–4 h). This can probably be attributed to steric reasons due to the greater difficulty of the branched oxyanion to approach FAFS’ terminal double bond as opposed to the less hindered fluorinated oxyanions. The yield of the branched allyl ether is also lower, 49% *vs.* 54%–86% for the linear perfluorinated oxyanions.

## 3. Experimental

### 3.1. General

^19^F-NMR spectra were recorded on a Varian Mercury 200 MHz spectrometer using CFCl_3_ as internal standard. The error on the measurement of the integrated intensities was ±5%. FT-IR spectra were recorded on a Nicolet Avatar 360 FT-IR ESP interfaced with OMINC software. Gas chromatographic analyses were performed on a Carlo Erba GC 8000 Top gas chromatographer using a silicone wide bore 0.54-micron thick 25 meters long column. Unless otherwise stated, all commercial reagents were used without further purification. All reported NMR chemical shifts are expressed in ppm.

*Caution!* Due to the high toxicity of SO_3_, BF_3_ and several monomers described hereforth, in particular ICF_2_CF=CF_2_, all reactions must be carried out in an efficient fume-hood wearing appropriate lab apparel.

### 3.2. Synthesis of CF_2_=CFCF_2_OSO_2_F (FAFS)

**Scheme 16:**
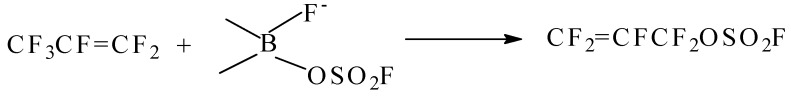
SO_3_-Boron complex in FAFS synthesis.

The following is a modified and revised procedure of FAFS [1,2,3,4,5]. Freshly distilled SO_3_ (50 g, 0.625 mol; b.p. = 43 °C) from 65% (w/w) oleum (Merck Industries) were placed in a glass Carius tube and connected to a BF_3_ bomb; 0.85 g of BF_3_ (1.7% w/w) were bubbled in the SO_3_ and dissolved with vigorous shaking. After 3 h a homogeneous, transparent and tanned colored solution is obtained. Care must be taken not to let T < 15 °C otherwise the irreversible SO_3_ polymerization will occur even in the presence of the BF_3_/SO_3_ complex (Scheme 1 and Scheme 16). The SO_3_ solution is transferred in a stainless steel 0.5 L autoclave, which is under vacuum. The autoclave is placed on a rocker at 25 °C and HFP (1.13 mol = 168.8 g) are pumped in the autoclave in 15–20 min. The temperature is raised to 37 °C for 12 h with constant rocking. The autoclave is then cooled to 0 °C, the excess HFP is evacuated and the crude, fuming reaction mixture is fractionally distilled.

CF_2_=CFCF_2_OSO_2_F is obtained in 67 mol % yield *vs.* SO_3_ (96 g; b.p. = 64 °C); ^19^F-NMR (CFCl_3_, std): +50 (s; 1F; –OSO_2_F); −71 (s; 2F; –CF_2_O–); −88; *^2^J_FF_* = 82, *^3^J_FF_* = 64 (dd; 1F; *cis* CF_2_=); −102.0; *^2^J_FF_* = 85, *^3^J_FF_* = 112 (ddt; 1F; *trans* CF_2_=); −190.5 (m; 1F; CF_2_=CF–); FT-IR (KBr): 1790 cm^−1^ (CF_2_=CFCF_2_–; st.); 1278 cm^−1^; 1166 cm^−1^; 1034 cm^−1^ (–CF–; st).

### 3.3. Synthesis of CF_2_=CFCF_2_OCH_3_—Without Basic Catalysis

CH_3_OH (15 g, 0.47 mol) are cooled to 0 °C with stirring; FAFS (8 g, 0.035 mol) are slowly added with a dropping funnel taking care not to exceed 15 °C. The reaction mixture is warmed to 20 °C and allowed to stir for 1 h. The crude mixture is washed twice with 30 mL H_2_O and dried over MgSO_4_. CF_2_=CFCF_2_OCH_3_ is obtained in 67 mol % yield (3.8 g) *vs.* FAFS. ^19^F-NMR (CFCl_3_, std): −73.5 (m; 2F; –CF_2_–O); −92.0; *^2^J_FF_* = 83, *^3^J_FF_* = 65 (dd; 1F; *cis* CF_2_=); −102.0; *^2^J_FF_* = 85, *^3^J_FF_* = 111 (ddt; 1F; *trans* CF_2_=); −189.0 (m; 1F; –CF_2_=CF–); ^1^H-NMR (TMS, std): 3.35 (s; 3H; CH_3_O–); FT-IR (KBr): 1785 cm^−1^ (CF_2_=CFCF_2_–; st); 1275 cm^−1^; 1157 cm^−1^; 1040 cm^−1^ (–CF–; st).

#### 3.3.1. Synthesis of CF_2_=CFCF_2_OCH_2_CF_3_—without basic catalysis

CF_3_CH_2_OH (13 g, 0.13 mol) was cooled to 0 °C with stirring; FAFS (6 g, 0.026 mol) was slowly added with a dropping funnel taking care not to exceed 15 °C. The reaction mixture is warmed to 20 °C and allowed to stir for 1 h. The crude mixture is washed twice with H_2_O (30 mL) and dried over MgSO_4_. CF_2_=CFCF_2_OCH_2_CH_3_ is obtained in 46 mol % yield (2.1 g) *vs*. FAFS.

#### 3.3.2. Synthesis of CF_2_=CFCF_2_OCH_2_CF_3_—with basic catalysis

CF_3_CH_2_OH (14 g, 0.14 mol) are added to KOH (1 g, 0.0178 mol) and mixed at 20 °C until a homogeneous solution is obtained. The mixture is cooled to 0 °C and FAFS (6 g, 0.026 mol) is slowly added with a dropping funnel making sure not to exceed an internal temperature of 15 °C. The reaction mixture is warmed to 20 °C and let stir for 2 h. The crude mixture is the washed with H_2_O and the organic phase is dried over MgSO_4_. CF_2_=CFCF_2_OCH_2_CF_3_ is obtained in 75% yield (4.5 g) *vs.* FAFS. ^19^F-NMR (CFCl_3_, std): −73.2 (t; 3F; *J* = 13.2 Hz, 6.6 Hz; CF_3_–CH_2_–); −73.0 (m; 2F; –CF_2_–O); −92.5; *^2^J_FF_* = 82, *^3^J_FF_* = 63 (dd; 1F; *cis* CF_2_=); −104.5; *^2^J_FF_* = 83, *^3^J_FF_* = 112 (ddt; 1F; *trans* CF_2_=); −189.5 (m; 1F; –CF_2_=CF–); ^1^H-NMR (TMS, std): 4.4 (q; 2H; CF_3_CH_2_O–); FT-IR (KBr): 1790 cm^−1^ (CF_2_=CFCF_2_–; st); 1275 cm^−1^; 1166 cm^−1^; 1040 cm^−1^ (–CF–; st).

### 3.4. Synthesis of CF_2_=CFCF_2_OC_6_F_5_, CF_2_=CFCF_2_OC_6_H_5_ and CF_2_=CFCF_2_OCH_2_C_6_H_5_

The following detailed procedure is for CF_2_=CFCF_2_OC_6_F_5_. The same procedure and molar quantities were employed for CF_2_=CFCF_2_OC_6_H_5_ and CF_2_=CFCF_2_OCH_2_C_6_H_5_*.* A heterogeneous mixture of NaH (2.76 g, 115 mmol) and anhydrous THF (20 mL) was cooled to 15 °C and stirred for 30 min. The mixture is cooled further to 4 °C and C_6_F_5_OH (20.1 g, 109 mmol) diluted in anhydrous THF (50 mL) are dripped in at a rate of 10 mmol/min. The reaction is exothermic (+20 °C) and its completion (10 min) is monitored by observing the ^19^F-NMR shift of the *para* F from –171 ppm (C_6_F_5_OH) to –187 ppm (C_6_F_5_O^−^Na^+^). FAFS (25 g, 109 mmol) is slowly added making sure not to exceed an internal temperature of 30 °C. After 60 min the reaction is complete and FAFS conversion = 100% as evidenced by ^19^F-NMR. The crude mixture is first filtered separating FSO_3_Na (13.5 g) and then distilled. CF_2_=CFCF_2_OC_6_F_5_ is obtained in 56% isolated yield (18.2 g, 61 mmol), b.p. = 57 °C at 14 mm Hg = 160 °C at 760 mm Hg. CF_2_=CFCF_2_OC_6_H_5_ is obtained in 87% isolated yield (21.2 g, 94.8 mmol). CF_2_=CFCF_2_OCH_2_C_6_H_5_ is obtained in 50% isolated yield (12.9 g; 54.5 mmol).

*CF_2_=CFCF_2_OC_6_F_5_*: ^19^F-NMR (CFCl_3_, std): −70.2 (m; 2F; –CF_2_–O); −88.5; *^2^J_FF_* = 83, *^3^J_FF_* = 64 (dd; 1F; *cis* CF_2_=); −102.0; *^2^J_FF_* = 85, *^3^J_FF_* = 110 (ddt; 1F; *trans* CF_2_=); −150.7 (m; 2F; *ortho*–);−154.6 (t; 1F; *para*–); −160.6 ppm (t; 2F; *meta*–); −188.9 ppm (m; 1F; CF_2_=CF–); FT-IR (KBr): 1785 cm^−1^(CF_2_=CFCF_2_–; st); 1625 cm^−1^; 1525 cm^−1^ (–C=C–; st; Ar); 1250 cm^−1^; 1155 cm^−1^; 1015 cm^−1^ (–CF–; st).

*CF_2_=CFCF_2_OC_6_H_5_*: ^19^F-NMR (CFCl_3_, std): −70.1 (m; 2F; –CF_2_–O); −90.5; *^2^J_FF_* = 83, *^3^J_FF_* = 64 (dd; 1F; *cis* CF_2_=); −103.5; *^2^J_FF_* = 85, *^3^J_FF_* = 110 (ddt; 1F; *trans* CF_2_=); −187.4 (m; 1F; CF_2_=CF–); FT-IR (KBr): 1789 cm^−1^ (CF_2_=CFCF_2_–; st); 1535 cm^−1^ (–C=C–; st; Ar), 1270 cm^−1^; 1150 cm^−1^; 1035 cm^−1^ (–CF–; st); ^1^H-NMR (TMS, std): 7.35, 7.2, 7.1 (m; 2H:1H:2H; –OC_6_H_5_).

*CF_2_=CFCF_2_OCH_2_C_6_H_5_*: ^19^F-NMR (CFCl_3_, std): −70.0 (m; 2F; –CF_2_–O); −92.5; *^2^J_FF_* = 81, *^3^J_FF_* = 63 (dd; 1F; *cis* CF_2_=); −104.5; *^2^J_FF_* = 83, *^3^J_FF_* = 111 (ddt; 1F; *trans* CF_2_=); −187.0 (m; 1F; CF_2_=CF–); FT-IR (KBr): 2985 cm^−1^ (–CH_2_O–Ar; st); 1789 cm^−1^ (CF_2_=CFCF_2_–; st.); 1590 cm^−1^; 1480 cm^−1^ (–C=C–; st; Ar); 1270 cm^−1^; 1150 cm^−1^; 1035 cm^−1^ (–CF–; st); ^1^H-NMR (TMS, std): 7.15, 7.1, 7.0 (m; 2H:1H:2H; –OCH_2_C_6_H_5_); 4.4 (–OCH_2_–Ar).

### 3.5. Synthesis of 2,4-Dinitrophenyl Perfluoroallyl Ether; CF_2_=CFCF_2_OC_6_H_3_(NO_2_)_2_ and p-Nitro Phenyl Perfluoroallylether CF_2_=CFCF_2_OC_6_H_4_(NO_2_)

The following detailed procedure is for CF_2_=CFCF_2_OC_6_H_3_(NO_2_)_2_. The same procedure and molar quantities were adopted for CF_2_=CFCF_2_OC_6_H_4_(NO_2_). A heterogeneous mixture of NaH (1.5 g, 63 mmol) and anhydrous CH_3_CN (20 mL) was cooled to 15 °C and stirred for 30 min. The mixture is cooled further to 5 °C and C_6_H_3_(NO_2_)_2_OH, (10 g, 53 mmol) dissolved in anhydrous CH_3_CN (95 mL) was dripped in at a rate of 10 mmol/min. The reaction is exothermic and care was taken to not exceed an internal temperature of 10 °C. At the end of the exotherm, phenate formation was complete and FAFS (12.5 g, 54 mmol) were added at a rate of 20 mmol/min. The reaction is modestly (+2 °C) exothermic. After 3 h the reaction was stopped. FAFS conversion = 81% (pushing the conversion lowered selectivity from 85% to 78%). The crude mixture was first filtered to remove FSO_3_Na, then washed with aqueous Na_2_CO_3_ (pH = 10, 200 mL) and finally flash chromatographed on silica gel eluting with CH_2_Cl_2_. CF_2_=CFCF_2_OC_6_H_3_(NO_2_)_2_ is obtained in 55% yield (9.3 g, 29.7 mmol). CF_2_=CFCF_2_OC_6_H_4_(NO_2_) is obtained in 75% isolated yield (10.9 g, 40.5 mmol).

*CF_2_=CFCF_2_OC_6_H_3_(NO_2_)_2_*: ^19^F-NMR (CFCl_3_, std): −70.0 (m; 2F; –OCF_2_CF=); −91.0; *^2^J_FF_* = 81, *^3^J_FF_* = 65 (dd; 1F; *cis* CF_2_=); −103.7; *^2^J_FF_* = 82, *^3^J_FF_* = 113 (ddt; 1F; *trans* CF_2_=); −191 (m; 1F; CF_2_=CF–); ^1^H-NMR (TMS, std): 8.55 (m; 1H; –C(NO_2_)=CHC(NO_2_)–); 8.32 (m; 1H; –OC=CH–CH=C(NO_2_)–); 7.6 (m; 1H; OC=CH–CH=C(NO_2_)–); FT-IR (KBr): 1520 cm^−1^ (symm.; Ar–NO_2_; st); 1345 cm^−1^ (asymm.; Ar–NO_2_; st); 1789 cm^−1^ (CF_2_=CFCF_2_–; st); 1600 cm^−1^; 1450 cm^−1^ (–C=C–; st; Ar); 1270 cm^−1^; 1150 cm^−1^; 1035 cm^−1^ (–CF–; st).

*CF_2_=CFCF_2_OC_6_H_4_(NO_2_)*: ^19^F-NMR (CFCl_3_, std): −70.1 (m; 2F; –OCF_2_CF=); −91.2; *^2^J_FF_* = 83, *^3^J_FF_* = 64 (dd; 1F; *cis* CF_2_=); −102.5; *^2^J_FF_* = 85, *^3^J_FF_* = 110 (ddt; 1F; *trans* CF_2_=); −189.4 (m; 1F; CF_2_=CF–); ^1^H-NMR (TMS, std): 8.47 (m; 2H; =CH–C(NO_2_)=CH–); 7.6 (m; 2H; =CH–C(OR_f_)=CH–); FT-IR (KBr): 1525 cm^−1^ (symm.; Ar–NO_2_; st); 1335 cm^−1^ (asymm.; Ar–NO_2_; st); 1789 cm^−1^(CF_2_=CFCF_2_–; st); 1620 cm^−1^; 1440 cm^−1^ (–C=C–; st; Ar); 1270 cm^−1^; 1150 cm^−1^; 1035 cm^−1^ (–CF–; st).

### 3.6. Synthesis of m-Cresol Perfluoroallylether CF_2_=CFCF_2_OC_6_H_4_–CH_3_

A heterogeneous mixture of NaH (1.5 g, 63 mmol) and anhydrous CH_3_CN (20 mL) was cooled to 15 °C and stirred for 30 min. The mixture is cooled further to 5 °C and a solution of *m*-C_6_H_4_CH_3_OH, (6.48 g, 60 mmol) in anhydrous CH_3_CN (75 mL) was dripped in at a rate of 10 mmol/min. The reaction is exothermic and care was taken to not exceed an internal temperature of 10 °C. At the end of the exotherm, phenate formation was complete and FAFS (13.8 g, 60 mmol) were added at a rate of 20 mmol/min. The reaction is modestly (+2 °C) exothermic. After 3 h the reaction was stopped. The crude mixture was first filtered to remove FSO_3_Na, then washed with aqueous Na_2_CO_3_ (pH = 10, 200 mL) and finally flash chromatographed on silica gel eluting with CH_2_Cl_2_. CF_2_=CFCF_2_OC_6_H_4_CH_3_ is obtained in 48% yield (5.9 g; 24.8 mmol). ^19^F-NMR (CFCl_3_, std): −61 (s; 3F; Ar–CF_3_); −68.7 (m; 2F; –OCF_2_CF=); −91.3; *^2^J_FF_* = 82, *^3^J_FF_* = 63 (dd; 1F; *cis* CF_2_=); −103.7; *^2^J_FF_* = 83, *^3^J_FF_* = 110 (ddt; 1F; *trans* CF_2_=); −188.5 (m; 1F; CF_2_=CF–); FT-IR (KBr): 2995 cm^−^^1^ (CH_3_–Ar; st); 1792 cm^−^^1^ (CF_2_=CFCF_2_–; st); 1580 cm^−^^1^; 1380 (–C=C–; st; Ar); 1275 cm^−^^1^; 1156 cm^−^^1^; 1030 cm^−^^1^ (–CF–; st).

### 3.7. Synthesis of CF_3_OCF_2_CF=CF_2_

Anhydrous KF (1.7 g; 30.3 mmol; 800 ppm residual H_2_O) was placed in a stainless steel autoclave. The autoclave is evacuated and cooled to –100 °C. Anhydrous diglyme (20 mL; 55 ppm residual H_2_O) and COF_2_ (2.3 g; 35 mmol) are condensed in the autoclave which is then warmed to 5 °C. The mixture is magnetically stirred at 1,000 rpm for 2 h in order to form the alcoholate. FAFS (6.7 g; 29 mmol) was then added from a pressurized (He; 7 atm) cylinder. The reaction is kept stirring at 5 °C for 1 h and 4 h at 20 °C. The crude mixture is then distilled directly from the autoclave under reduced pressure. The fraction boiling at 11 °C was identified as CF_3_OCF_2_CF=CF_2_ (3.38 g, 15.7 mmol). Isolated yield = 54% *vs.* FAFS. ^19^F-NMR (CFCl_3_, std): −53.5 (s; 3F; CF_3_O–); −71.7 (m; 2F; –OCF_2_CF=); −87.8; *^2^J_FF_* = 82, *^3^J_FF_* = 65 (dd; 1F; *cis* CF_2_=); −101.3; *^2^J_FF_* = 83, *^3^J_FF_* = 111 (ddt; 1F; *trans* CF_2_=); −190.3 (m; 1F; CF_2_=CF–); FT-IR (KBr): 1787 cm^−1^ (CF_2_=CFCF_2_–; st); 1270 cm^−1^; 1156 cm^−1^; 1025 cm^−1^ (–CF–; st).

### 3.8. Synthesis of CF_3_CF_2_OCF_2_CF=CF_2_

Anhydrous KF (36.5 g; 630 mmol) are placed in a glass round bottomed flask equipped with a condenser (−78 °C), a magnetic stir bar, a dropping funnel and a thermometer. Anhydrous diglyme (400 mL) is added along with CF_3_COF (78 g; 670 mmol; b.p. = −56 °C) previously condensed in an Erlenmeyer flask. The reaction flask is warmed to 5 °C and stirred for 1 h. FAFS (150 g; 630 mmol) is then slowly added taking care not to exceed 10 °C inside the flask. The reaction is let stir at 5 °C for 1 h and then 4.5 h at 20 °C. Already after 1 h at 20 °C the crude mixture separates into two phases. The product is distilled and 142 g of the fraction boiling at 39–40 °C were collected and identified as CF_3_CF_2_OCF_2_CF=CF_2_. Yield = 86%. ^19^F-NMR (CFCl_3_, std): −86 (m; 2F; CF_3_CF_2_–O–); −84.3 (s; 3F; CF_3_–); −69.5 (m; 2F; –OCF_2_CF=); −87.5; *^2^J_FF_* = 83, *^3^J_FF_* = 64 (dd; 1F; *cis* CF_2_=); −101; *^2^J_FF_* = 85, *^3^J_FF_* = 110 (ddt; 1F; *trans* CF_2_=); −189.3 (m; 1F; CF_2_=CF–); FT-IR (KBr): 1792 cm^−1^ (CF_2_=CFCF_2_–; st); 1273 cm^−1^; 1186 cm^−1^; 1027 cm^−^^1^ (–CF–; st).

### 3.9. Synthesis of CF_3_CF_2_CF_2_OCF_2_CF=CF_2_

Anhydrous KF (1.4 g; 24 mmol) was placed in a glass round bottomed flask equipped with a condenser (−78 °C), a magnetic stir bar, a dropping funnel and a thermometer. Anhydrous diglyme (18 mL) was added along with CF_3_CF_2_COF (4.1 g; 24 mmol), previously condensed in a Carius tube. The reaction flask is warmed to 5 °C and stirred for 1 h. FAFS (6 g; 26 mmol) are then slowly added taking care not to exceed 10 °C inside the flask. The reaction is allowed to stir at 5 °C for 1 h and then 3 h at 20 °C. Already after 1 h at 20 °C the crude mixture separates into two phases. The product is distilled and 6 g of the fraction boiling at 47–49 °C were collected and identified as CF_3_CF_2_CF_2_OCF_2_CF=CF_2_. Yield = 84%. ^19^F-NMR (CFCl_3_, std): −71.5 (m; 2F; =CFCF_2_–O); −81.5 (s; 3F; CF_3_–); −84.7 (s; 2F; CF_3_CF_2_CF_2_O–); −89.7; *^2^J_FF_* = 81, *^3^J_FF_* = 65 (dd; 1F; *cis* CF_2_=); −103.1; *^2^J_FF_* = 83, *^3^J_FF_* = 112 (ddt; 1F; *trans* CF_2_=); −130.2 (s; 2F; CF_3_CF_2_CF_2_O–); −192.5 (m; 1F; CF_2_=CF–); FT-IR (KBr): 1788 cm^−1^ (CF_2_=CFCF_2_–; st); 1270 cm^−1^; 1150 cm^−1^; 1145 cm^−1^; 1035 cm^−1^ (–CF–; st).

### 3.10. Synthesis of FSO_2_CF_2_CF_2_OCF_2_CF=CF_2_

Anhydrous KF (1.1 g; 19.6 mmol) and anhydrous diglyme (3 mL) were placed in a glass round bottom flask equipped with a condenser (−10 °C), a magnetic stir bar, a dropping funnel and a thermometer. FSO_2_CF_2_COF (3.3 g; 18.3 mmol; b.p. = 28 °C) was added directly from the stainless steel cylinder with a PTFE steel-glass connector. The mixture is stirred at 0 °C for 45 min and then FAFS (4.3 g; 18.7mmol) was slowly added taking care not to exceed an internal temperature of 10 °C. The mixture is stirred at 1,000 rpm for 3 h during which time FSO_3_K is formed. The crude mixture is distilled and the fraction boiling at 105 °C was identified as FSO_2_CF_2_CF_2_OCF_2_CF=CF_2_ (5.1 g). Yield = 84%. ^19^F-NMR (CFCl_3_, std): +46 (s; 1F; –SO_2_F); −111 (s; 2F; FSO_2_CF_2_–); −81 (s; 2F; FSO_2_CF_2_CF_2_O–); −90; *^2^J_FF_* = 83, *^3^J_FF_* = 64 (dd; 1F; *cis* CF_2_=); −103; *^2^J_FF_* = 85, *^3^J_FF_* = 110 (ddt; 1F; *trans* CF_2_=); −70 (s; 2F; –OCF_2_CF=); −190 (m; 1F; CF_2_=CF–); FT-IR (KBr): 1792 cm^−1^ (CF_2_=CFCF_2_–; st); 1275 cm^−1^; 1160 cm^−1^; 1041 cm^−1^ (–CF–; st).

### 3.11. Synthesis of CF_2_=CFCF_2_O-OCF_2_CF=CF_2_

#### 3.11.1. Aqueous H_2_O_2_ route (Trial 33; Table 11)

NaOH (0.19 g, 4.8 mmol) was dissolved in H_2_O (2 mL) and placed in a glass round bottom flask equipped with a “Micro-mix” mechanical stirrer, a dropping funnel, a condenser (−78 °C) and a thermometer. Care is taken to treat all glassware with dichromate solution prior to performing the reaction in order to eliminate all possible organic residues that may decompose the peroxides. The mixture is cooled to 0 °C and stirred at 750 rpm. Aqueous H_2_O_2_ (30% w/w; 240 μL, 2.39 mmol 100% H_2_O_2_) is added with a micro-syringe and the mixture is stirred at 0 °C for 5 min. FAFS (1.0 g, 4.35 mmol) are dripped in every 5–10 seconds. There is an immediate temperature increase; the maximum internal temperature was 8 °C (T_MAX_), which was reached in 6 min. After T_MAX_, the internal temperature dropped to 2 °C in 3 min. The peroxidation reaction is over in a total reaction time of 9 min. The crude mixture is immediately separated in a pre-chilled separation funnel collecting the lower, organic phase (not a clear-cut separation), which was placed in an NMR tube thermostated at 20 °C for kinetic measurements. FAFS conversion = 100%; CF_2_=CFCF_2_O–OCF_2_CF=CF_2_ yield = selectivity = 26.7%.

#### 3.11.2. Anhydrous H_2_O_2_ route

CaH_2_ (0.44 g, 10.56 mmol) dispersed in CH_3_CN (3 mL) was placed in a glass round bottom flask equipped with a “Micro-mix” mechanical stirrer, a dropping funnel, a condenser (−78 °C) and a thermometer. Care is taken to treat all glassware with dichromate solution prior to performing the reaction in order to eliminate all possible organic residues that may decompose the peroxides. The dispersion is stirred at 750 rpm at 20 °C for 30 min. The apparatus is then fluxed with N_2_ (5 L/h) and then H_2_O_2_ (30% w/w; 0.271 g, 2.39 mmol H_2_O_2_ 100%; 10.56 mmol H_2_O) is added quickly. No exothermicity was observed. FAFS (1.0 g; 4.35 mmol), previously diluted in anhydrous CH_3_CN (0.5 mL) is quickly added. The reaction is exothermic and reached T_MAX_ = 27 °C in 5 min. In order to contain the reaction exothermicity, the reaction was periodically dipped in an ethanol/dry ice bath at –15 °C. The reaction temperature returned to 0 °C in 10 min and was kept stirring at 0 °C for 30 min. The reaction was then warmed to 20 °C and stirred for an additional 2 h. The crude reaction mixture was filtered to separate Ca(OH)_2_ obtaining a colorless, clear solution. CF_2_=CFCF_2_O–O–CF_2_CF=CF_2_ yield = 32%.

^19^F-NMR (CFCl_3_, std):

*CF_2_=CFCF_2_O–OCF_2_CF=CF_2_*: −81.4 (m; 4F; –O–OCF_2_–); −89.5 (m; 2F; –CF_2_=CF–); −104.3 (m; 2F; –CF_2_=CF–); −188.8 (m; 2F; –CF_2_=CF–).

*CF_2_=CFC(=O)O–OC(=O)CF=CF_2_*: −80.6 (dd; 2F; –CF_2_=CF–); −92.6 (ddt; 2F; –CF_2_=CF–); −186.4 (m; 2F; –CF_2_=CF–).

*HOOCCFHCOOH*: −192.2 (d; 1F; –CFH–; *J_F,H_* = 56 Hz).

*HOOCCFHC(=O)O–OC(=O)CFHCOOH*: −195.2 (d; 2F; –CFH; *J_F,H_* = 56 Hz).

*CF_3_CFHCOOH:* −86.6 (m; 3F; CF_3_–); −200.8 (dm; 1F; –CFH; *J_F,H_* = 56 Hz).

*CF_3_CFHC(=O)O–O–C(=O)CFHCF_3_*: −86.9 (m; 6F; CF_3_–); −204.6 (dm; 2F;–C_F_H–; *J_F,H_* = 56 Hz).

*CF_2_=CFC(=O)–O–OH*: −82.3 (dd; 1F; CF_2_=CF–); −93.1 (dd; 1F; CF_2_=CF–); −182.4 (dd; 1F; CF_2_=CF–).

*CF_3_CFHC(=O)*–*O*–*OH*: −86.3 (m; 3F; CF_3_–); −200.6 (dm; 1F; –C_F_H–; *J_F,H_* = 55 Hz).

*HOOCCFHC(=O)O*–*OH*: −191.7 (d; 1F; –CFH–; *J_F,H_* = 50 Hz).

*CF_2_=CFCOOH*: −82.9 (dd; 1F; CF_2_=CF–); −93.5 (dd; 1F; CF_2_=CF–); −182.2 (dd; 1F; CF_2_=CF–).

### 3.12. Synthesis of (CF_3_)_2_CFCF(CF_3_)O–CF_2_CF=CF_2_

#### 3.12.1. Synthesis of (CF_3_)_2_CFC(=O)CF_3_

Anhydrous KF (2.0 g, 34 mmol) and anhydrous diglyme (20 mL) are placed in a stainless steel autoclave equipped with a magnetic stir bar and a pressure transducer. The autoclave is first evacuated and cooled to –100 °C and then CF_3_C(=O)F (20 g, 172 mmol) and HFP (25.8 g, 172 mmol) are condensed in the autoclave. The autoclave is heated to 100–110 °C and stirred at 1,000 rpm for 8 h. The autoclave is cooled to 20 °C and the residual pressure of unreacted reagents is slowly bleeded away. The crude diglyme mixture is first filtered to remove KF and then distilled. The fraction boiling at 30–35 °C was identified as (CF_3_)_2_CFC(=O)CF_3_. Isolated yield = 70% (32 g; 120 mmol). ^19^F-NMR (CFCl_3_, std): −74.4 (m; 6F; (CF_3_)_2_CF–); −76.1 (m; 3F; CF_3_C(=O)–); −192.5 (h; 1F (CF_3_)_2_CF–).

#### 3.12.2. Synthesis of (CF_3_)_2_CFCF(CF_3_)O–CF_2_CF=CF_2_

Anhydrous KF (2.18 g, 37.5 mmol) was suspended in anhydrous diglyme (15 mL) and stirred at 1,000 rpm for 15 min at 0 °C. (CF_3_)_2_CFC(=O)CF_3_ (10 g, 37.6 mmol) was added within 10 min and alloed to stir for 3 h. FAFS (9.2 g, 40 mmol) was added in 15 min and the reaction mixture is stirred at 0 °C for 4 h and then warmed to 10 °C and stirred for an additional 8 h. The crude mixture was filtered to remove FSO_3_K and then washed twice with distilled H_2_O. Yield = 49% (7.7 g). ^19^F-NMR (CFCl_3_, std): −67.6 (m; 2F; =CFCF_2_–O); −70.0 (dm; 6F; (CF_3_)CF–) (m; 3F; –OCF(CF_3_)–); −92 (m; 1F; *J_F,F_* = 48 Hz; CF_2_=); −104.4 (m; 1F; *J_F,F_* = 117 Hz, 27 Hz; CF_2_=); −133.2 (q; 1F; *J_F,F_* = 17 Hz, –OCF(CF_3_)–CF–); −182.0 (h; 1F; (CF_3_)_2_CF–); −189 (dm; 1F; *J_F,F_* = 40 Hz, 118 Hz; CF_2_=CF–CF_2_–); FT-IR (KBr): 1791.5 cm^−1^ (CF_2_=CFCF_2_–; st); 1250 cm^−1^; 1222 cm^−1^; 1084 cm^−1^; 1013 cm^−1^ (–CF–; st).

### 3.13. Synthesis of CF_2_=CFCF_2_N_3_

NaN_3_ (2.82 g, 43.4 mmol) was suspended in anhydrous CH_3_CN (10 mL) and stirred at 20 °C for 15 min. FAFS (10 g, 43.5 mmol) is added in 5 min and the mixture was stirred at 20 °C for 3 h. The mixture was filtered and analyzed. FAFS conversion = 98%; CF_2_=CFCF_2_N_3_: selectivity = 74%. Non-isolated yield 72%. CAUTION! The allyl azide could be explosive [25]. ^19^F-NMR (CFCl_3_, std): −76.0 (m; 2F; –NCF_2_CF=); −92.5; *^2^J_FF_* = 82, *^3^J_FF_* = 63 (dd; 1F; *cis* CF_2_=); −105.0; *^2^J_FF_* = 85, *^3^J_FF_* = 112 (ddt; 1F; *trans* CF_2_=); −190.0 (m; 1F; CF_2_=CFCF_2_O–).

### 3.14. Synthesis of ICF_2_CF=CF_2_

Anhydrous KI (1.52 g, 9.13 mmol) was suspended in CH_3_CN (5.0 mL) and anhydrous DMF (0.1 mL) in a glass round bottom flask equipped with a dripping funnel, a magnetic stir bar, a condenser (10 °C) and a thermometer. The heterogeneous mixture is cooled to 0 °C with stirring (750 rpm). FAFS (2.0 g, 8.69 mmol) was added in 3 min. The maximum exothermicity observed was +4 °C after 10 min. After 3 h at 0 °C FAFS conversion = 100%. The crude mixture is filtered and distilled. The fraction boiling at 41 °C was identified as ICF_2_CF=CF_2_ (1.85 g, 7.1 mmol). Yield = 82%. BrCF_2_CF=CF_2 _and ClCF_2_CF=CF_2_ were synthesized in an analogous manner.

*ICF_2_CF=CF_2_*: ^19^F-NMR (CFCl_3_, std): −48 (t; 2F; –CF_2_I); −92; *^2^J_FF_* = 83, *^3^J_FF_* = 65 (dd; 1F; *cis* CF_2_=); −102.0; *^2^J_FF_* = 85, *^3^J_FF_* = 110 (ddt; 1F; *trans* CF_2_=); −175 (m; 1F; CF_2_=CF–).

*BrCF_2_CF=CF_2_*: ^19^F-NMR (CFCl_3_, std): −58.2 (t; 2F; –CF_2_Br); −96; *^2^J_FF_* = 84, *^3^J_FF_* = 66 (dd; 1F; *cis* CF_2_=); −106.3; *^2^J_FF_* = 85, *^3^J_FF_* = 111 (ddt; 1F; *trans* CF_2_=); −186 (m; 1F; CF_2_=CF–).

*ClCF_2_CF=CF_2_*: ^19^F-NMR (CFCl_3_, std): −77 (t; 2F; –CF_2_Cl); −93, *^2^J_FF_* = 84, *^3^J_FF_* = 67 (dd; 1F; *cis* CF_2_=); −104.5; *^2^J_FF_* = 83, *^3^J_FF_* = 112 (ddt; 1F; *trans* CF_2_=); (m; 1F; CF_2_=); −186.5 (m; 1F; CF_2_=CF–).

## 4. Conclusions

FAFS was demonstrated to be an easily synthesizable, extremely versatile and useful monomer for preparing a wide selection of perfluoroallyl monomers such as fluorinated or partially fluorinated aromatic and aliphatic allyl ethers, allyl halides, diallyl-alkyl peroxides and allyl azides respectively from readily available alcohols, phenols, acyl fluorides, ketones, metal halides, H_2_O_2_ and sodium azide.

According to the conditions employed, FAFS can be directed to perform Addition/Elimination reactions versus Substitution reactions yielding respectively perfluoroallyl ethers and perfluoroallyl sulfate esters. These novel allylic compounds have the potential of becoming useful co-monomers or modifying agents for fluoropolymers.
